# Factors associated with parents' self-reported familiarity with pediatric eye disorders in Jordan: a quantitative analysis of survey data from Jordanian parents

**DOI:** 10.3389/fmed.2026.1935571

**Published:** 2026-09-11

**Authors:** Asma Musleh, Omar Bdair, Ayman A. Abdul Aziz, Amal F. Alomari, Sara Issa, Mohammad Abusamak, Almutez Gharaibeh, Abdalla Al-Jazzazi, Layan Alhaj Ali, Yara Bdair, Bashar Majali, Rima Al-Dabbas, Zaid Al-Abbadi, Ruba Salameh, Mira Salman, Mona Freihat

**Affiliations:** 1 Department of Special Surgery, Faculty of Medicine, Al-Balqa Applied University, Al-Salt, Jordan; 2 Faculty of Engineering Technology, Al-Balqa Applied University, Amman, Jordan; 3 Faculty of Medicine, Memorial University of Newfoundland, St. John’s, NL, Canada; 4 Department of Special Surgery, The University of Jordan, Amman, Jordan; 5 Brother Rice Junior High, St. John’s, NL, Canada; 6 Ophthalmology Department, Jordanian Ministry of Health, Amman, Jordan; 7 Ophthalmology Department, University Hospital Leicester NHS Trust, Leicester, United Kingdom

**Keywords:** cross-sectional survey, health literacy, Jordan, parental awareness, pediatric eye disorders, self-reported familiarity

## Abstract

**Introduction:**

Parents play an important role in recognizing potential eye problems in children, yet their knowledge and perceptions regarding pediatric eye disorders remain insufficiently characterized in Jordan. This study assessed several dimensions of parental eye-health awareness and examined factors associated with parents' self-reported familiarity with common pediatric eye disorders.

**Methods:**

We performed a cross-sectional survey on 1,264 parents residing in all twelve governorates of Jordan. The questionnaire had five domains: knowledge of pediatric eye diseases, recognition of symptoms and causes, treatment and management, attitudes toward eye care, and barriers to accessing services. We also asked the parents to classify their familiarity with common pediatric eye disorders, and we used this as the main study outcome. We analyzed the data using descriptive statistics, Wilcoxon signed-rank tests, independent-samples *t*-tests, and binary logistic regression.

**Results:**

It was found that most of the participants were women (70.7%), married (93.7%), and had a bachelor's degree (54.7%). 61.1% of parents classified themselves as familiar with common pediatric eye disorders. Over the five domains, parental attitudes toward eye care had the highest mean score, followed by recognition of symptoms and causes. Parental attitudes toward eye care had the highest overall domain score, followed by recognition of symptoms and causes. Participants reporting familiarity had significantly higher scores than those reporting no familiarity across all five domains. In the domain-level regression analysis, only knowledge of pediatric eye diseases (OR = 2.13, *p* < 0.001) and treatment and management (OR = 1.97, *p* < 0.001) remained independently associated with self-reported familiarity. Over all domains' items, the strongest predictors were: correct knowledge about the age range affected by amblyopia, awareness that strabismus can lead to amblyopia, and correct understanding that wearing glasses does not worsen a child's vision.

**Conclusions:**

Participating parents generally demonstrated positive attitudes toward pediatric eye care but showed important gaps in disease-specific and treatment-related knowledge. Several knowledge and treatment-related items were associated with self-reported familiarity, even after adjustment for participant characteristics. These findings identify potential targets for parental eye-health education but should not be interpreted as evidence of actual care-seeking behavior, treatment adherence, or clinical decision-making.

## Introduction

Clear vision is essential for children's healthy development, personality formation, and interaction with the world around them. Undiagnosed or untreated vision problems in children not only affect eye health but also impact a child's learning abilities, cognitive development, self-confidence, and social interaction. In the long term, these problems can have social and economic consequences (1). The World Health Organization's report on vision emphasized these points and highlighted the importance of good vision for facilitating a child's access to education and enabling them to reach their full potential (2). Children with visual impairment, living in low- and middle-income countries, suffer the consequences of vision problems, having up to five times less chance of attending formal education compared to their peers with good vision (3). The global scope of this problem cannot be ignored. 19 million children under the age of 14 were considered to have visual impairment in 2010 (4). Another study to review the prevalence of refractive errors across the world, reviewing international databases from 1990 to 2016, found that the estimated pool prevalence of myopia, hypermetropia and astigmatism in children was 11.7%, 4.6% and 14.9%, respectively (5). Refractive errors constitute the largest share of vision problems, while amblyopia (lazy eye) and strabismus account for approximately 2%–4% (6). The alarming aspect is that most of these cases could have been either prevented or at least treated if diagnosed early. Problems such as anisometropic amblyopia, for example, significantly improved when the underlying cause was treated in children under 7 years old (7), and the same applies to other conditions such as strabismus (crossed eyes), refractive errors, and others (8). This is where the role of parents becomes crucial, especially in the absence of government programs to screen all children, particularly in low-income and resource-scarce countries. Parents are usually the first to notice that something may be wrong with their child's vision. For example, a child may squint frequently, tilt the head, sit unusually close to the television, or begin to have difficulty at school. When parents recognize such symptoms, they may seek an eye examination and obtain further medical advice. On the other hand, when parents fail to recognize these symptoms or think they are unrelated to vision problems, assessment may be delayed and the child may miss the opportunity for early intervention. As a result, this will negatively impact the child's vision and future development. One study showed that parents' unawareness of the causes of common pediatric eye disorders has negatively affected their response and behaviour to seek specialized health care for their children (9). The level of health literacy among parents and its impact on their behavior regarding their children's health is an important topic that requires greater attention and focus. It is estimated that 25% of parents have limited health awareness in terms of disease prevention, going through to the treatment and follow-up of chronic pediatric conditions, which leads to poor health outcomes in their children (10). In the context of eye health specifically, there are a considerable number of misconceptions and false beliefs among parents. For example, a study showed that a substantial number of parents fear that the glasses prescribed to correct their children's vision may be unnecessary and could even weaken their vision in the future (11). Such misconceptions may influence treatment acceptance and adherence, as suggested by previous studies. Correcting inaccurate beliefs may therefore represent an important component of parental eye-health education. Published studies on parental awareness of children's eye problems have increased significantly in recent years, despite the uneven geographical distribution of the studied areas. A study conducted in Mecca (KSA) concluded that parents had an overall low level of knowledge and urged the establishment of structured programs to increase awareness in this area (12). A study conducted in Al Madina (KSA) also demonstrated the unsatisfactory level of knowledge among parents (13). Another study showed the key barriers limiting access to the specialized eye care included insufficient parents' knowledge and gaps in school-based screening programs (14). In Jordan, this topic has not received much attention, and most studies measuring knowledge of eye diseases have focused on adult eye conditions. Other studies have focused on the knowledge level of healthcare providers rather than parents. A nationwide survey aimed at studying public awareness of common eye diseases such as diabetes, cataracts, and glaucoma in adults, revealed that the level of awareness is relatively good and comparable to that in developed countries (15). Another study published in 2021, assessing the knowledge and attitudes of pediatricians in northern Jordan regarding common childhood eye diseases, revealed that only about 10% perform routine eye examinations as part of a child's general health check-up, and only 62% knew that refractive errors can appear at any age (16). The few available studies from Jordan show a gap in awareness, knowledge, and practices related to dealing with pediatric eye disorders. These gaps not only affect the general population but also the healthcare professionals involved in child health. In particular, there is limited information on what parents know about children's eye problems, how they recognize possible eye problems in their children, and the difficulties they may face when trying to obtain appropriate eye care.

Research on parents' awareness of childhood eye conditions has increased in nearby countries, but information from Jordan remains limited. In particular, little is known about what Jordanian parents understand about pediatric eye diseases, the symptoms they associate with these conditions, available treatment options, their attitudes toward eye care, and the barriers they may experience when seeking services for their children.

Based on these gaps, the present study has two aims. First, we examined and compared responses across five areas of pediatric eye health: disease knowledge, recognition of symptoms and causes, treatment and management, attitudes toward eye care, and perceived barriers to accessing care. Second, we identified which domain-level and item-level factors were associated with parents' self-reported familiarity with common pediatric eye disorders. In this study, self-reported familiarity refers to the participant's personal perception and should not be considered a measure of actual care-seeking, treatment adherence, or clinical decision-making.

## Methods

### Design and setting

A cross-sectional survey was conducted among parents and guardians living in Jordan. Participants were recruited from all 12 governorates, with responses obtained from both urban and rural areas. The cross-sectional design was used to describe participants' knowledge, attitudes, and other questionnaire responses within a defined period of time (17). This broad geographic coverage aimed to capture responses from parents with different social, educational, and regional backgrounds.

### Participants and recruitment

Any parent or guardian of a child under 18 years of age living in Jordan during the survey period was eligible to participate in the study. Because eligibility also included guardians, reporting no children under 18 did not necessarily indicate absence of a caregiving role. The study was open to all parents without any restrictions related to gender, age, occupation, educational level, or place of residence. Social media platforms were employed to distribute the questionnaire. Recruitment represented a non-probability, self-selected online sampling approach rather than population-based random sampling. Although this method facilitated participation from parents across all 12 governorates, individuals who regularly use social media or who have a greater interest in children's health may have been more likely to encounter and participate. This approach was used to facilitate broad geographic recruitment and reach parents from different regions of Jordan (18). Potential participants who accessed the invitation to participate post were directed to electronically sign a consent to participate, which was followed by the survey itself. Participants were assured of confidentiality, that their participation was voluntary, and that they could withdraw from the study at any time without any consequences to minimize any tendency toward socially desirable responding.

G*Power (version 3.1) was used to determine the required sample, applying the following parameters: effect size of 0.3, statistical power of 0.9, and alpha level of 0.05 (19). The final sample included 1,264 participants, exceeding the required minimum and considered sufficient for the planned domain-level and item-level regression analyses.

### Questionnaire development, validation, and data collection

The research team prepared the questionnaire after reviewing published work on parents’ awareness and knowledge of pediatric eye health, including studies from Arab countries and other settings (9, 11–13, 20, 21). The topics covered in these studies were used as a guide when choosing items for the present questionnaire. Items considered relevant to the Jordanian setting were adapted where needed, and similar questions were combined. Some wording was also simplified so that the questionnaire could be understood by parents with different educational and regional backgrounds.

The items were grouped into five domains according to the topic they addressed: knowledge of pediatric eye diseases, recognition of symptoms and causes, treatment and management, parental attitudes toward eye care, and barriers to accessing eye-care services. These domains were defined based on the clinical content of the questions and the main areas the study intended to examine.

Before approving and distributing the questionnaire, it was reviewed by three pediatric ophthalmologists. They examined the questions for clarity, clinical relevance, terminology, and whether the main aspects of pediatric eye health were adequately covered. Their comments led mainly to minor changes in wording and organization. The revised version was then confirmed via pilot testing by 20 parents who met the eligibility criteria. These parents were asked whether the questions were easy to understand and complete and whether the questionnaire was too demanding. No major problems were identified during this pilot stage, and no substantial changes were made before the full survey was launched, as the pilot feedback indicated that the questions were clear and understandable, and no further changes were required.

The questionnaire consisted of two sections. The first collected demographic and background information. It also included the question whether parents considered themselves familiar with common pediatric eye problems: “Are you familiar with common pediatric eye disorders?” Participants answered Yes or No. In the analyses, this item was used as the primary outcome variable in the regression analyses. This response represented the participant's own view of how familiar they were with pediatric eye conditions; it was not treated as an objective measure of knowledge. This may influence how they interpret symptoms, seek information, and engage with healthcare services. A person answering “Yes” could be influenced by several factors, including previous exposure to information about eye health, confidence in their own knowledge, professional experience, or previous contact with pediatric eye-care services. For this reason, we refer to this measure throughout the study as self-reported familiarity.

Knowledge and treatment-related understanding were examined separately through the remaining questionnaire items. The second section included five domains. Responses were recorded on a three-point scale: 1 = Disagree, 2 = Neutral, and 3 = Agree. This format was retained after the pilot participants indicated that they found it straightforward to use. Participants found this format easier to understand and complete and reported less hesitation. Because the survey was intended for parents with different educational backgrounds, we preferred a simple response format that could be completed easily and directly by potential participants. Although scales with five or seven response options allow finer distinctions between levels of agreement, ease of understanding and completion was given greater priority in this survey. The five domains of this study covered knowledge of pediatric eye diseases (6 items), recognition of eye disease symptoms and causes (9 items), treatment and management of eye disorders (10 items), parental attitudes toward eye health care (9 items), and barriers to accessing eye care (8 items).

It is worth noting here that the barriers domain differs in interpretation from the other domains. For other domains, higher scores indicate better knowledge or more positive attitudes. In contrast, the barriers domain was phrased so that agreement reflected fewer barriers to care, while disagreement indicated a greater burden of barriers. For example, a statement such as “*Financial constraints, such as doctor's fees and the cost of glasses or surgery, do not prevent me from getting eye care for my child*” would receive a lower score from a parent who experiences financial difficulty. For this, a lower mean score in this domain reflects a higher perceived burden of barriers. The direction of these items was considered when analysing the domain scores and in the interpretation process (22). In particular, lower scores were treated as indicating a greater perceived burden of barriers. The complete questionnaire is provided as Supplementary Material.

### Data analysis

IBM SPSS (Version 23) was used for the statistical analysis of the data. Internal consistency was assessed using Cronbach's alpha for the complete 42-item questionnaire and separately for each of the five domains. Domain-specific coefficients were calculated because the domains addressed different aspects of parental knowledge, attitudes, treatment awareness, and barriers to care. The score for each domain was calculated by finding the arithmetic mean of the domain's constituent items, while maintaining a 3-point scale, which allowed for direct comparison across all domains. Missing data were not replaced or imputed. Descriptive and bivariate analyses used all available observations for the variables involved in each analysis. Multivariable models and analyses that required simultaneous measurements used complete-case analysis. Thus, a participant was excluded from a particular multivariable analysis only when data were missing for one or more variables required by that model. Because the number of variables differed across analyses, the corresponding analytic sample sizes also differed. The number of participants included in each major analysis is reported in the Results and relevant tables. Of the 1,264 participants, five had no valid response for the primary self-reported familiarity outcome, leaving 1,259 participants available for analyses involving this outcome. Complete scores for all five questionnaire domains were available for 1,258 participants.

Descriptive summaries were prepared for the sample and for individual questionnaire items. These included frequencies and percentages for categorical responses, together with means, standard deviations, modes, and mode percentages. Domain scores were described using means, standard deviations, medians, and mean ranks. Since the five domain scores were obtained from the same participants, their distributions were compared using the Friedman test. Kendall's *W* was used as an overall effect-size measure. When the Friedman test indicated a significant difference, the domains were compared in pairs using Wilcoxon signed-rank tests. Ten pairwise comparisons were possible using Bonferroni corrections. Thus, the adjusted Bonferroni significance threshold was set at p<0.005. These analyses were intended to describe relative response patterns across the domains and not to compare the scores with an externally validated clinical cutoff. This non-parametric approach was chosen after the Kolmogorov–Smirnov and Shapiro–Wilk tests confirmed that none of the domain scores followed a normal distribution (*p* < 0.05 for all), consistent with standard practice in survey-based health research (23). Between-group comparisons of domain scores by familiarity status were conducted using independent samples *t*-tests, with results cross-validated against non-parametric alternatives where normality was in question. The dependent variable represented self-reported or perceived familiarity rather than objectively verified knowledge. Familiarity was coded as 1 for parents who answered Yes and 0 for those who answered No.

Binary logistic regression was performed to identify factors associated with parents’ self-reported familiarity with common pediatric eye disorders. At the domain level, all five prespecified domain scores were entered in the first step. Backward elimination based on the Wald statistic was then used to obtain a final model. This procedure was considered appropriate for the domain-level analysis because it involved only five conceptually defined predictors rather than a large set of individual candidate variables. Because the primary outcome was self-reported familiarity, some conceptual overlap with the knowledge and treatment-related questionnaire domains was expected. The regression analyses were therefore used to identify questionnaire characteristics associated with participants' perception of familiarity rather than to establish independent prediction of objectively measured knowledge or healthcare behavior.

Before fitting the domain-level model, multicollinearity among the five domain scores was assessed using tolerance and variance inflation factors. Tolerance values above 0.20 and VIF values below 5 were considered to indicate the absence of concerning multicollinearity. Model fit was evaluated using the −2 log likelihood, Cox and Snell $R^{2}$, Nagelkerke $R^{2}$, and the Hosmer–Lemeshow goodness-of-fit test.

The item-level analysis was conducted in two stages. First, backward elimination was applied to the 42 questionnaire items as an exploratory screening procedure to identify a smaller set of candidate item-level predictors. Second, the items retained in the exploratory model were entered simultaneously using the ENTER method into an adjusted logistic regression model that also included educational level, monthly income, medical background, previous diagnosis of an eye disorder in a participant's child, sex, and urban or rural residence. All adjustment variables were retained in the adjusted model irrespective of their statistical significance. Odds ratios, adjusted odds ratios, 95% confidence intervals, and two-sided *p*-values were reported, as appropriate.

### Ethical considerations

The study was conducted in accordance with the Declaration of Helsinki. The required ethical approvals were obtained from the Medical Ethics Council of Al-Balqa Applied University, As Salt, Jordan (No. 2025/2024/7/1). All participants electronically signed a consent form agreeing to the use of their data for the study's purposes before accessing the study questionnaire. It was clearly stated that participation was voluntary, informed about the purpose of the study, and the provided information was guaranteed to be confidential. No personal questions were asked at any stage of the survey.

## Results

Out of all people who received the survey, 1,264 participants were included in the analysis who met the conditions and agreed to participate.

### Missing data and analytic sample sizes

The overall study sample consisted of 1,264 participants. Missing data were limited but varied across individual variables. The primary self-reported familiarity outcome was available for 1,259 participants (773 familiar and 486 not familiar), with five missing responses. Information regarding previous diagnosis of an eye disorder in a child was available for 1,256 participants, with eight missing responses. Complete scores across all five questionnaire domains were available for 1,258 participants. No imputation was performed.

Consequently, analytic sample sizes varied according to the variables required for each analysis. The Friedman comparison of the five domain scores included the 1,258 participants with complete domain data. The exploratory item-level logistic regression, which simultaneously entered all 42 questionnaire items, was based on 1,043 complete cases because SPSS applied listwise exclusion when any item required by the model was missing. The subsequent adjusted item-level model included 1,218 participants with complete information for the five retained questionnaire items and the prespecified demographic and background covariates.

### Sociodemographic characteristics

The sociodemographic characteristics of the participants are presented in Table 1. Women represented 70.7% of participants (n=894), while men accounted for 29.3% (n=370). Most respondents were married (93.7%, n=1,184); the remaining 6.4% were either divorced or widowed. Participants were recruited from all 12 governorates. The largest proportion came from Amman (42.2%, *n* = 533), followed by Balqa (18.0%, *n* = 228), Zarqa (12.1%, *n* = 153), and Irbid (8.7%, *n* = 110). Representation from the other governorates ranged from 1.3% to 6.2%. Most participants lived in urban areas (82.8%, n=1,047), compared with 17.2% (n=217) from rural areas.

**Table 1:** Sociodemographic characteristics of participants (*n* = 1,264).

Characteristic	*n*	%
Gender		
Male	370	29.3
Female	894	70.7
Marital status		
Married	1,184	93.7
Divorced	40	3.2
Widow	40	3.2
Province		
Amman	533	42.2
Balqa	228	18.0
Zarqa	153	12.1
Irbid	110	8.7
Madaba	78	6.2
Mafraq	35	2.8
Karak	25	2.0
Ajloun	23	1.8
Ma'an	22	1.7
Jarash	21	1.7
Aqaba	19	1.5
Tafila	17	1.3
Area of residence		
Urban	1,047	82.8
Rural	217	17.2
Educational level		
No education	5	0.4
Elementary school	16	1.3
High school	149	11.8
Diploma	167	13.2
Bachelor	691	54.7
Master	152	12.0
PhD	84	6.6
Employment status		
Student	52	4.1
Medical field worker	156	12.3
Governmental employee	336	26.6
Private employee	202	16.0
Business owner	67	5.3
Retired	138	10.9
Unemployed	313	24.8
Monthly income (JD)		
<500	486	38.4
500–1,000	509	40.3
1,000–2,000	168	13.3
>2,000	101	8.0
Number of children <18 years		
0	197	15.6
1	247	19.5
2	316	25.0
3	262	20.7
4	165	13.1
Child diagnosed with eye disorder		
Yes	512	40.5
No	744	58.9

More than half of the participants had a bachelor's degree (54.7%, n=691). A further 13.2% (n=167) reported a diploma, 12.0% (n=152) a master's degree, 6.6% (n=84) a PhD, and a high school education (11.8%, n=149). Only a small number had elementary education (1.3%, n=16) or no formal education (0.4%, n=5).

Government employees formed the largest occupational group (26.6%), followed by participants who were unemployed (24.8%). Private-sector employees accounted for 16.0% of the sample, and 12.3% worked in the medical field. Retired participants represented 10.9%, while business owners and students made up 5.3% and 4.1%, respectively. Monthly income was below JD 500 for 38.4% of respondents and between JD 500 and JD 1,000 for 40.3%; only 8.0% reported an income above JD 2,000 per month. Regarding children under 18 years of age, 19.5% reported one child, 25.0% reported two, and 20.7% reported three children. It was found that 40.5% of the families had a child previously diagnosed with an eye condition.

For the main outcome of this study, 61.1% considered themselves familiar with the common pediatric eye disorders, while 38.9% were not. This finding reflects perceived familiarity and should not be interpreted as the proportion of parents who demonstrated objectively adequate knowledge. The participants were divided into two groups, namely familiar and unfamiliar, which is the basis of all subsequent group comparisons and regression analyses.

### Questionnaire reliability

The overall 42-item questionnaire showed good internal consistency, with a Cronbach's alpha of 0.820. Domain-specific coefficients varied: 0.504 for knowledge of pediatric eye diseases, 0.625 for recognition of symptoms and causes, 0.572 for treatment and management, 0.755 for parental attitudes toward eye care, and 0.825 for barriers to eye care. Thus, internal consistency was strongest for the attitudes and barriers domains and lower for the domains assessing disease-specific knowledge and treatment. The lower coefficients may reflect the breadth of the clinical content covered by these domains, in which individual items assess different diseases, symptoms, and treatment concepts rather than closely interchangeable aspects of a single narrow construct.

### Item-level descriptive statistics

Table 2 has the mean, standard deviation, modes, and mode percentages for the 42 questionnaire items. The range of mean scores for these items was from 1.7 to 2.88, which reflects the degree of variation in individual responses across the sample.

**Table 2:** Item-level descriptive statistics for decision-making domains (*n* = 1,258).

Item	Description	Mean	SD	Mode	Mode %
Domain 1: knowledge of pediatric eye diseases					
Domain1_1	Amblyopia can affect children up to 7–8 years old	2.37	0.69	3	48.7
Domain1_2	A child could have refractive error without complaining of blurry vision	2.41	0.76	3	58.1
Domain1_3	Delay in treatment of refractive errors could lead to amblyopia	2.69	0.57	3	73.8
Domain1_4	Strabismus in children could lead to amblyopia	2.51	0.68	3	61.3
Domain1_5	It is abnormal to see strabismus in a child up to 1 year old	1.74	0.83	1	50.8
Domain1_6	Eye surgeries, when recommended, are necessary for children's vision health	2.73	0.56	3	78.4
Domain 2: recognition of eye disease symptoms and causes					
Domain2_1	Children who struggle to copy from the board or perform poorly in school should have an eye examination	2.83	0.46	3	85.7
Domain2_2	Headaches in children can result from eye problems	2.80	0.49	3	83.5
Domain2_3	Frequent rubbing of the eye indicates an eye problem	2.76	0.52	3	79.7
Domain2_4	Poor focus in a child could be due to an eye problem	2.65	0.61	3	71.8
Domain2_5	Frequent blinking, light sensitivity and squeezing of the eyes indicate need for an eye exam	2.86	0.41	3	88.4
Domain2_6	Eye pain, watering and discharge are not always caused by eye infection	1.70	0.81	1	51.9
Domain2_7	Difficulty identifying colors in a child could indicate an eye disorder	2.73	0.57	3	78.3
Domain2_8	Tilting the head frequently could indicate an eye condition	2.57	0.65	3	65.4
Domain2_9	Avoiding close objects or toys might indicate an eye problem	2.44	0.71	3	56.1
Domain 3: treatment and management of eye disorders					
Domain3_1	Wearing prescribed glasses helps children develop normal vision	2.81	0.49	3	85.0
Domain3_2	Wearing glasses all the time does not make children dependent on them or worsen their vision	2.36	0.81	3	57.2
Domain3_3	Amblyopia can be treated depending on the child's age	1.78	0.77	1	43.5
Domain3_4	Amblyopia can be treated by surgeries and/or glasses and/or eye drops with covering the good eye	2.00	0.78	2	38.5
Domain3_5	It is not true to ignore lacrimal obstruction until school age	2.30	0.75	3	47.9
Domain3_6	Treatment of strabismus includes glasses and surgery	2.65	0.60	3	71.8
Domain3_7	Correcting strabismus surgically does not eliminate the need to wear glasses	1.93	0.77	2	39.3
Domain3_8	Using eye drops without consulting a doctor is risky for children	2.86	0.43	3	88.8
Domain3_9	Strabismus in children is not treated for cosmetic reasons only	2.72	0.60	3	79.4
Domain3_10	Eye disorders like cataract and glaucoma could affect old people and children	2.08	0.86	3	41.8
Domain 4: parental attitudes toward eye health and care					
Domain4_1	All children should have a routine eye exam even if they show no signs of vision problems	2.83	0.45	3	85.2
Domain4_2	The first routine eye exam with no vision complaints should be done at age 3–5 years	2.64	0.64	3	72.0
Domain4_3	School vision screening is not sufficient to detect all vision problems	2.52	0.77	3	68.5
Domain4_4	A child with family history of eye disorders should have an eye examination even without complaints	2.81	0.48	3	83.9
Domain4_5	Regular eye exams every 6–12 months are necessary for children's eye health	2.67	0.60	3	73.1
Domain4_6	Parents should follow the doctor's recommended treatment even if they believe the child doesn't need it	2.85	0.44	3	87.3
Domain4_7	If the teacher or pediatrician recommends an eye examination, parents should follow their recommendation	2.88	0.38	3	89.4
Domain4_8	Previously prescribed eye drops to one child should not be used on another without consulting the doctor	2.82	0.50	3	86.4
Domain4_9	Using electronic devices and screens for a long time increases the possibility of myopia and eye strain	2.87	0.42	3	89.9
Domain 5: barriers to eye care^a^					
Domain5_1	Financial constraints do not prevent me from getting eye care for my child	1.96	0.89	1	41.7
Domain5_2	Distance to the nearest eye doctor does not make it difficult to access eye care	2.13	0.89	3	46.0
Domain5_3	The need for several doctor visits and waiting times does not affect my commitment to an eye test	2.00	0.90	3	40.4
Domain5_4	Difficulties in eye testing do not affect my decisions regarding my child's eye health	2.23	0.85	3	49.8
Domain5_5	Concerns about cosmetic appearance of glasses or bullying do not discourage me from seeking care	2.51	0.77	3	67.1
Domain5_6	Concerns about surgery outcomes or anesthesia complications do not affect my agreement to surgery	2.20	0.86	3	49.2
Domain5_7	Recommendations from family and friends cannot affect my decisions regarding my child's eye health	2.49	0.77	3	65.2
Domain5_8	Lack of trust in health care providers does not influence my decisions regarding my children's eye health	1.91	0.88	1	42.6

a Domain 5 items are reverse-coded; a lower mean reflects greater perceived barriers to eye care.

In Domain 1, the highest mean was observed for the statement that eye surgery, when recommended, may be necessary for children's visual health (Item 1_6; mean = 2.73), followed by awareness that delayed treatment of refractive errors could lead to amblyopia (Item 1_3; mean = 2.69). The lowest score was observed for the statement that strabismus in a child younger than 1 year is abnormal (Item 1_5; mean = 1.74), indicating uncertainty or misunderstanding regarding early childhood strabismus.

In Domain 2, the highest score was observed for the item concerning frequent blinking, light sensitivity, or forceful eye squeezing as signs that a child may need an eye examination (Item 2_5; mean = 2.86; modal response = 3, 88.4%). A similarly high score was recorded for the statement that children who have difficulty copying from the board or perform poorly at school should undergo an eye examination (Item 2_1; mean = 2.83). In contrast, Item 2_6 had the lowest mean in this domain (mean = 1.70; mode = 1). This item stated that eye pain, tearing, and discharge are not always caused by an eye infection. The pattern of responses suggests that many participants may have difficulty distinguishing infectious causes from other possible causes of these symptoms. Such misunderstanding could potentially affect how parents interpret these symptoms and when they seek professional assessment.

In Domain 3, it was noticed that parents strongly agreed that the use of eye drops without medical advice can be harmful to children (Item 3_8; mean = 2.86). The parents also strongly agreed that prescribed glasses support normal visual development (Item 3_1; mean = 2.81). However, the parents also showed noticeable gaps in understanding of some treatment approaches used for children's eye disorders. For example, (Item 3_3) recorded a mean of just 1.78 with a modal disagreement response, as many parents believed that amblyopia can be treated depending on age. (Item 3_5) recorded a mean of 2.30, as many parents believed that blocked tear ducts should not be ignored until school age. Similarly, items addressing the complexity of amblyopia treatment (Item 3_4; mean = 2.00), as well as the belief that glasses were necessary after strabismus surgery (Item 3_7; mean = 1.93). This reflects a lack of awareness and knowledge regarding the treatments available for childhood eye diseases.

For Domain 4, the results showed strong awareness and positive attitudes regarding following the guidelines of official authorities when it comes to making decisions related to children's eye health. Agreement was high across all nine domain items, achieving means above 2.50 and modal responses of 3. The highest score was for the item related to seeking an eye examination from a specialist based on recommendations from a teacher or pediatrician (Item 4_7; mean = 2.88), followed by the item related to electronic-device/screen use increases myopia and eye strain and adhering to the doctor's recommendations and treatments, even if parents are uncertain (Item 4_9; mean = 2.87) and (Item 4_6; mean = 2.85), respectively.

In Domain 5, which studies barriers to accessing medical care, interpreted in light of their reverse-coded phrasing. Financial constraints were found to be the biggest obstacle (Item 5_1; mean = 1.96, mode = 1, 41.7%), followed closely by a lack of trust in healthcare providers (Item 5_8; mean = 1.91, mode = 1, 42.6%). These two items scored the lowest means across the domain, suggesting that a significant proportion of parents considered them the most important factors preventing their children from receiving the necessary care. Other commonly reported barriers included geographical access (Item 5_2; mean = 2.13), the burden of repeated clinic visits and waiting times (Item 5_3; mean = 2.00), and concerns about possible complications related to surgery (Item 5_6; mean = 2.20). In comparison, concerns about the appearance of eyeglasses (Item 5_5; mean = 2.51) and the influence of relatives or friends (Item 5_7; mean = 2.49) appeared to be less prominent barriers.

### Comparison of scores across domains

Table 3 summarizes Domain score comparisons. Complete scores for all five questionnaire domains were available for 1,258 participants. Parental attitudes toward eye care had the highest mean score and mean rank (mean = 2.77, SD = 0.31; mean rank = 4.31), followed by recognition of symptoms and causes (mean = 2.59, SD = 0.30; mean rank = 3.44). Knowledge of pediatric eye diseases had a mean score of 2.41 (SD = 0.33; mean rank = 2.63), while treatment and management had a mean score of 2.35 (SD = 0.32; mean rank = 2.42). The barriers-to-care domain had the lowest mean score and rank (mean = 2.18, SD = 0.57; mean rank = 2.20). Because higher scores in the barriers domain reflected fewer barriers, its lower score indicated a greater perceived burden of barriers.

**Table 3:** Descriptive scores and relative ranking across the five questionnaire domains (n=1,258).

Domain	Mean	SD	Median	Mean rank
Knowledge of pediatric eye diseases	2.41	0.33	2.50	2.63
Recognition of symptoms and causes	2.59	0.30	2.67	3.44
Treatment and management	2.35	0.32	2.40	2.42
Parental attitudes toward eye care	2.77	0.31	2.89	4.31
Barriers to eye care^a^	2.18	0.57	2.25	2.20

a Domain 5 items are reverse-coded; a lower mean reflects greater perceived barriers to eye care.

The Friedman test showed that the domain-score distributions differed significantly, $\chi^{2}(4)=1,588.67,\;p<0.001$. Kendall's *W* was 0.316, indicating a moderate overall difference among the domain scores. Bonferroni-adjusted Wilcoxon signed-rank tests showed significant differences between all ten pairs of domains (p<0.001 for each comparison). These findings describe the relative pattern of responses across the five domains and do not indicate whether any domain reached a clinically validated level of adequate knowledge, attitudes, or access to care.

### Domain score differences by familiarity status

Domain scores were compared between participants who reported familiarity with common pediatric eye disorders and those who did not (Table 4). Participants reporting familiarity had significantly higher scores across all five domains. The largest absolute difference was observed for knowledge of pediatric eye diseases, with familiar participants having a mean score of 2.44 (SD = 0.32) compared with 2.35 (SD = 0.34) among those reporting no familiarity (*p* < .001). Recognition of symptoms and causes was also higher among familiar participants [2.62 (SD = 0.29) vs. 2.55 (SD = 0.31)], (*p* < .001).

**Table 4:** Domain score differences by self-reported parental familiarity status.

Domain	Familiar *n*	Mean (SD)	Not familiar *n*	Mean (SD)	*t* (df)	*p*
Knowledge of pediatric eye diseases	773	2.44 (0.32)	486	2.35 (0.34)	5.07 (1,257)	<.001
Recognition of symptoms and causes	773	2.62 (0.29)	486	2.55 (0.31)	3.63 (1,257)	<.001
Treatment and management	773	2.38 (0.34)	486	2.30 (0.28)	4.81 (1,174.88)^a^	<.001
Parental attitudes toward eye care	772	2.78 (0.29)	485	2.74 (0.33)	2.09 (931.70)^a^	.037
Barriers to eye care^b^	770	2.21 (0.57)	483	2.13 (0.56)	2.50 (1,251)	.013
Overall composite score	773	2.49 (0.24)	486	2.42 (0.22)	5.26 (1,257)	<.001

a Welch's *t*-test reported because the homogeneity-of-variance assumption was not met.

b Higher scores indicate fewer perceived barriers; therefore, the higher score among familiar participants indicates fewer perceived barriers.

For treatment and management, familiar participants had a mean score of 2.38 (SD = 0.34), compared with 2.30 (SD = 0.28) among non-familiar participants. Because the homogeneity-of-variance assumption was not met, the Welch test was used, (*p* < .001). Parental attitudes toward eye care were also slightly higher among familiar participants [2.78 (SD = 0.29)] than among non-familiar participants [2.74 (SD = 0.33)], (*p* = .037).

The barriers-to-care score was higher among familiar participants [2.21 (SD = 0.57)] than among non-familiar participants [2.13 (SD = 0.56)], (*p* = .013). Because higher scores in this domain indicate fewer perceived barriers, this result suggests that participants reporting familiarity also reported a somewhat lower perceived burden of barriers. The overall composite score was similarly higher among familiar participants [2.49 (SD = 0.24)] than among non-familiar participants [2.42 (SD = 0.22)], (*p* < .001).

### Domain-level factors associated with self-reported parental familiarity

Multicollinearity among the five domain scores was examined before fitting the logistic regression model. Tolerance values ranged from 0.719 to 0.862, while VIF values ranged from 1.160 to 1.392. These findings indicated no concerning multicollinearity among the domain scores. The final backward model showed acceptable calibration according to the Hosmer–Lemeshow test, $\chi^{2}(8)=8.43,\;p=0.392$. The final model had a −2 log likelihood of 1,632.97, a Cox and Snell $R^{2}$ of 0.030, and a Nagelkerke $R^{2}$ of 0.040.

The backward stepwise approach was employed in the domain-level binary logistic regression analysis. The results of the final model are summarized in Table 5. It was found that there was a significant positive association between knowledge of pediatric eye diseases and parental self-reported familiarity (*B* = 0.756, Wald = 16.66, *p* < 0.001; OR = 2.13, 95% CI: 1.48–3.06). This indicates that a higher score in this domain was linked to a greater likelihood of parents' group who considered themselves familiar with pediatric eye disorders. It was also found that treatment and management awareness was a significant predictor (*B* = 0.676, Wald = 12.74, *p* < 0.001; OR = 1.97, 95% CI: 1.36–2.85). The other domains did not remain significant, and they were excluded from the final model.

**Table 5:** Domain-level binary logistic regression—final model predicting parental familiarity (step 5).

Predictor	*B*	SE	Wald	df	*p*-value	OR	95% CI
Domain 1: knowledge of eye diseases	0.756	0.185	16.66	1	<0.001	2.13	1.48–3.06
Domain 3: treatment and management	0.676	0.189	12.74	1	<0.001	1.97	1.36–2.85
Constant	−2.930	0.566	26.83	1	<0.001	0.053	–

### Item-level factors associated with self-reported parental familiarity

At the item level, exploratory backward elimination was initially used in the logistic regression model, which started with all 42 questionnaire items to identify a smaller set of candidate item-level factors associated with self-reported familiarity. The final exploratory model retained five items as independent predictors of parental self-reported familiarity (Table 6).

**Table 6:** Exploratory item-level logistic regression examining factors associated with self-reported parental familiarity (step 38).

Item	Domain	*B*	SE	Wald	df	*p*-value	OR	95% CI
Amblyopia can affect children up to 7–8 years old	Knowledge	0.364	0.093	15.25	1	<0.001	1.44	1.20–1.72
Strabismus in children could lead to amblyopia	Knowledge	0.309	0.096	10.25	1	0.001	1.36	1.13–1.65
Wearing glasses does not worsen vision or cause dependence	Treatment	0.281	0.082	11.60	1	0.001	1.32	1.13–1.55
Multiple visits and waiting times do not affect eye care decisions^a^	Barriers	0.218	0.084	6.72	1	0.010	1.24	1.05–1.47
Procedural difficulties do not affect eye care decisions^a^	Barriers	−0.184	0.090	4.19	1	0.041	0.83	0.70–0.99
Constant		−1.868	0.404	21.42	1	<0.001	0.154	–

a Domain 5 items are reverse-coded; agreement reflects the absence of the stated barrier.

Two of these items came from the knowledge domain. Parents who agreed that amblyopia can affect children up to 7–8 years of age were more likely to report self-reported familiarity with pediatric eye disorders (OR = 1.44, 95% CI: 1.20–1.72, *p* < 0.001). A similar pattern was observed for the item stating that strabismus in children can lead to amblyopia (OR = 1.36, 95% CI: 1.13–1.65, *p* = 0.001). Another significant predictor came from the treatment domain: parents who agreed that wearing glasses continuously does not make children dependent on them and does not worsen their vision were also more likely to describe themselves as familiar (OR = 1.32, 95% CI: 1.13–1.55, *p* = 0.001).

The final model also retained two items from the barriers domain. Because this domain was coded in the opposite direction from the others, these findings should be interpreted with care. Parents who agreed that multiple clinic visits and long waiting times would not affect their decision of obtaining an eye examination had higher odds of being from the familiar group of parents (OR = 1.24, 95% CI: 1.05–1.47, *p* = 0.010). This may reflect a greater commitment from parents in seeking health care for their children in need. On the contrary, a significant association was found between parents who agreed that practical difficulties during the examination process would not affect decision-making and the lower odds of self-reported familiarity (OR = 0.83, 95% CI: 0.70–0.99, *p* = 0.041). Further consideration of this unexpected result is presented in the Discussion section.

Because participant characteristics could confound these item-level associations, all five retained items were subsequently examined in an adjusted model that included prespecified demographic and background variables.

### Adjusted item-level factors associated with self-reported familiarity

To examine whether the item-level findings were independent of participant characteristics, the five items retained in the original item-level model were entered into a second logistic regression model together with educational level, medical background, previous diagnosis of an eye disorder in a child, monthly income, sex, and area of residence. All covariates were retained in the model regardless of their statistical significance because they were entered in advance as potential confounders. The adjusted findings are presented in Table 7.

**Table 7:** Adjusted item-level binary logistic regression examining factors associated with self-reported parental familiarity with pediatric eye disorders.

Predictor	*B*	SE	Wald *χ*^2^	Adjusted OR	95% CI	*p*-value
Questionnaire items						
Amblyopia can affect children up to 7–8 years old	0.300	0.088	11.558	1.350	1.135–1.604	0.001
Strabismus in children could lead to amblyopia	0.247	0.090	7.608	1.280	1.074–1.526	0.006
Wearing glasses does not worsen vision or cause dependence	0.271	0.077	12.275	1.312	1.127–1.527	<0.001
Multiple visits and waiting times do not affect care decisions	0.181	0.078	5.396	1.198	1.029–1.396	0.020
Procedural difficulties do not affect care decisions	−0.127	0.084	2.327	0.880	0.747–1.037	0.127
Monthly income, JD			9.048			0.029
<500 vs. >2,000	−0.795	0.282	7.969	0.452	0.260–0.784	0.005
500–1,000 vs. >2,000	−0.656	0.270	5.895	0.519	0.305–0.881	0.015
1,000–2,000 vs. >2,000	−0.805	0.294	7.495	0.447	0.251–0.796	0.006
Educational level			0.006			0.997
High school or below vs. postgraduate	0.017	0.238	0.005	1.017	0.639–1.620	0.943
Diploma/bachelor's vs. postgraduate	0.004	0.169	0.001	1.004	0.721–1.398	0.981
Female vs. male	0.164	0.138	1.410	1.178	0.899–1.545	0.235
Urban vs. rural residence	−0.105	0.162	0.416	0.901	0.655–1.238	0.519
No medical background vs. medical background	−0.328	0.203	2.599	0.721	0.484–1.073	0.107
No previous child eye diagnosis vs. previous diagnosis	−0.219	0.125	3.083	0.803	0.629–1.026	0.079

Four of the five questionnaire items remained significantly associated with self-reported parental familiarity. Parents who agreed that amblyopia can affect children up to 7–8 years of age had higher odds of reporting familiarity with pediatric eye disorders (aOR = 1.35, 95% CI: 1.14–1.60, *p* = 0.001). Familiarity was also positively associated with awareness that strabismus can lead to amblyopia (aOR = 1.28, 95% CI: 1.07–1.53, *p* = 0.006), understanding that wearing glasses does not worsen vision or cause dependence (aOR = 1.31, 95% CI: 1.13–1.53, *p* < 0.001), and agreement that repeated clinic visits and waiting times would not affect the decision to obtain an eye examination (aOR = 1.20, 95% CI: 1.03–1.40, *p* = 0.020).

The inverse association for procedural difficulties during eye examination was attenuated after adjustment and was no longer statistically significant (aOR = 0.88, 95% CI: 0.75–1.04, *p* = 0.127). This suggests that the association observed in the original item-level model may have been influenced by differences in participant characteristics.

Monthly income was significantly associated with self-reported familiarity overall, Wald *χ*^2^(3) = 9.05, *p* = 0.029. Compared with parents earning more than JD 2,000 per month, parents in each of the three lower-income groups had lower odds of reporting familiarity. Educational level, medical background, previous diagnosis of an eye disorder in a child, sex, and urban or rural residence were not independently associated with the outcome.

## Discussion

This study examined parents' self-reported familiarity with common pediatric eye disorders and how this perception was associated with knowledge, symptom recognition, treatment-related understanding, attitudes toward eye care, and perceived barriers. Parents with greater knowledge of specific pediatric eye conditions and better understanding of selected treatment issues were more likely to describe themselves as familiar. These findings should be interpreted as associations with perceived familiarity rather than evidence that these parents necessarily made different healthcare decisions or engaged in different care-seeking behaviors. Self-reported familiarity is closely related in concept to the questionnaire domains dealing with knowledge and treatment. Parents who considered themselves familiar with pediatric eye disorders would be expected to answer some of these questions differently from those who did not. The associations observed in the regression models may partly reflect this overlap rather than an entirely separate predictive relationship. Even so, the item-level results are useful because they show which specific aspects of knowledge and treatment understanding differed most clearly according to participants' reported familiarity.

Differences between the familiar and non-familiar groups were expected, but the pattern was not limited to one or two domains. In the bivariate analyses, participants who reported being familiar with pediatric eye disorders had higher scores in all five domains: knowledge, recognition of symptoms and causes, treatment and management, attitudes toward eye care, and barriers to care. When the five domains were entered together in the logistic regression model, only knowledge of pediatric eye diseases and treatment and management remained independently associated with self-reported familiarity. Thus, although the two groups differed across several areas, knowledge and treatment-related understanding showed the strongest independent relationships with the familiarity outcome.

### Six in ten parents reported familiarity—what does that mean?

In this study, 61.1% of participants considered themselves familiar with common pediatric eye disorders. This proportion is close to that reported in a Saudi Arabian study (24). However, the comparison should be interpreted cautiously because differences in study populations, sampling methods, and the way familiarity was assessed may influence the reported proportions. Importantly, this also means that around four in ten participants in the present study did not consider themselves familiar with common pediatric eye disorders.

That proportion should not be overlooked. Practically, it represents a substantial group of parents understands the importance of timely assessment or accept the treatment recommendations appropriately, even though they may not be able to recognize early signs of disease. This is important in countries with limited or no organized national screening programs for childhood eye conditions. In these countries, parents play an important role in linking the children with the health system. The finding also means that a substantial proportion of participants did not consider themselves familiar with common pediatric eye disorders. This perceived familiarity gap may be relevant to how parents interpret eye-health information or recognize the need for professional assessment. However, the present study did not measure actual care-seeking behavior, referral timing, treatment adherence, or clinical outcomes. The implications of lower perceived familiarity for these outcomes therefore require evaluation in prospective studies.

### Attitudes are an asset—but not sufficient on their own

Parental attitudes toward eye health care represented one of the strongest areas in the overall domain profile, with the attitudes domain having the highest mean score. Domain 4 achieved the highest composite mean of any domain (2.77). This reflects a near-universal consensus on following professional guidance: adhering to the recommendations of the teacher or pediatrician, following prescribed treatments, and attending follow-up clinic visits. The high attitudes-domain score indicates that participating respondents generally expressed favorable views toward professional eye care and following clinical recommendations. These responses should not, however, be interpreted as evidence that participants necessarily sought care, attended follow-up visits, or adhered to recommended treatment in practice. The problem is that being willing to seek care and knowing when to seek it are two different things. For example, a parent who agrees that their child should have a routine eye exam but doesn't know what amblyopia is will be unable to recognize its signs and symptoms. Similarly, misconceptions about glasses may influence treatment acceptance or care-seeking, as suggested by previous studies; however, these behaviors were not directly evaluated in the present study. This gap between knowledge and attitudes has been observed elsewhere in the region: in Saudi Arabia, more than four out of ten parents have never taken their children for an eye exam, with most justifying their actions by claiming that their children showed no signs of problems (13). From the above, it is clear that the absence of perceived need for the examination from the parents' point of view, which is primarily due to a lack of knowledge, was the main determining factor, and not the parents' unwillingness or negligence. Attitude scores were slightly but significantly higher among participants reporting familiarity than among those reporting no familiarity (2.78 vs. 2.74, *p* = .037). However, attitudes did not remain independently associated with familiarity when the questionnaire domains were considered simultaneously in the logistic regression model.

Taken together, these findings suggest that favorable attitudes and disease-specific knowledge represent related but distinct dimensions of parental eye-health awareness.

### What specifically distinguishes familiar from non-familiar parents

The exploratory item-level analysis originally identified five items that distinguished parents who reported familiarity with pediatric eye disorders from those who did not. These included two disease-knowledge items, one treatment-related item, and two items concerning barriers to care. Four of the five item-level associations remained statistically significant in the adjusted analysis. This model included the five selected questionnaire items together with educational level, medical background, previous diagnosis of an eye disorder in a child, monthly income, sex, and area of residence. The persistence of four associations after adjustment indicates that they were not fully accounted for by the differences in demographic and background characteristics of the participants.

Two of the retained items were related to amblyopia: knowing that amblyopia can affect children up to 7–8 years of age and recognizing that strabismus may lead to amblyopia. Both remained independently associated with self-reported familiarity after adjustment. These topics are clinically relevant because amblyopia develops during the period of visual maturation, and treatment is generally more effective when the condition is identified and managed during childhood (7). Parents who understand that the effective treatment period is limited may be better prepared to recognize the importance of early assessment and intervention. Future educational efforts should provide clear information about the relationship between strabismus and amblyopia. It should also emphasize that visible eye deviation in childhood should have medical assessment and not simply be expected to resolve on its own.

The treatment-related finding also remained significant after adjustment. Parents who correctly understood that prescribed glasses do not weaken children's vision or cause dependence were more likely to report familiarity with pediatric eye disorders. Misconceptions about glasses are important because they can affect treatment acceptance and adherence even when parents are otherwise willing to seek care. Similar concerns about eyeglasses have been documented in regional and international studies (11). Correct understanding of this issue may therefore represent more than knowledge of an isolated fact; it may indicate prior access to reliable eye-health information or previous communication with an eye-care professional.

Of the two barrier-related items identified in the exploratory analysis, only the item concerning repeated clinic visits and waiting times remained independently associated with familiarity. Parents who reported that these logistical demands would not affect their decision to obtain an eye examination had higher odds of reporting familiarity. This may indicate that parents who perceive themselves as more familiar with pediatric eye disorders are also more willing to tolerate the practical demands of seeking and continuing care. However, the cross-sectional design does not establish whether familiarity promotes commitment to care or whether previous engagement with eye-care services increases familiarity.

The second related item, concerning procedural difficulties during the eye examination, showed an inverse association with self-reported familiarity in the exploratory questionnaire-only model. However, this association was attenuated and was no longer statistically significant after adjustment for participant characteristics. The initial finding should therefore be interpreted cautiously and should not be considered an independent association. One possible explanation is that differences in previous healthcare experiences or familiarity with pediatric eye-examination procedures may have influenced participants' responses, although this cannot be determined from the present data. Further qualitative research may help clarify how parents perceive and respond to procedures used during pediatric eye examinations.

Monthly income also emerged as an independent factor in the adjusted model. Parents in each of the three lower-income categories had lower odds of reporting familiarity than parents earning more than JD 2,000 per month. This pattern may reflect differences in access to specialist consultations, reliable health information, and previous contact with eye-care services. It also indicates that educational efforts should pay particular attention to families with fewer financial resources. Nevertheless, because the highest-income reference group represented a relatively small proportion of the sample, the magnitude of these comparisons should be interpreted with appropriate caution.

Overall, the adjusted analysis narrows the most relevant intervention targets to a small number of actionable topics: the time-sensitive nature of amblyopia, its relationship with strabismus, misconceptions about prescribed glasses, and the practical burden of repeated visits and waiting times. These topics may therefore represent useful areas for future parental eye-health education. Whether educational interventions targeting these areas improve objective knowledge, care-seeking behavior, treatment adherence, or other healthcare outcomes should be evaluated prospectively.

### Recognition of symptoms differed by familiarity but did not remain independently associated

Recognition of eye disease symptoms and causes differed significantly according to self-reported familiarity. Participants reporting familiarity had a higher mean score in this domain than those reporting no familiarity (2.62 vs. 2.55, *p* < .001). However, the recognition-of-symptoms domain did not remain in the final domain-level logistic regression model when the five domains were considered simultaneously. Thus, although symptom recognition was associated with familiarity in the bivariate comparison, it did not show an independent association after accounting for the other questionnaire domains. This suggests that recognizing signs of eye disorders in children may be relatively common among parents, but this recognition does not necessarily translate into a deep understanding of pediatric eye disorders.

In practical terms, some parents may be able to notice abnormality with their children's eyes without knowing why it occurs, how serious it is, or whether urgent assessment matters. Therefore, the ability to recognize a symptom is different from understanding the implicit issue or abnormality. This distinction is important for community public health. Efforts that aim to increase public awareness of symptoms should be associated with clear information about the disorders and the importance of early evaluation. It may be helpful for parents to understand when and why action is needed if symptom recognition is connected with simple explanations.

### Barriers are real but operate differently than expected

Domain 5 had the lowest mean score among the five domains. Because the items in this domain were coded reversely, the results indicate that barriers to eye care were commonly reported by participants. Financial constraints and lack of trust in healthcare providers were among the most prominent concerns. In other words, many participants disagreed with statements suggesting that these factors would not interfere with obtaining eye care for their children. Similar concerns have been reported in other studies, where the costs of eyeglasses, medical visits, and surgical procedures were identified as important difficulties for low-income families. Lack of trust in healthcare providers has also been described as a potential barrier to accessing care (14, 21).

In the bivariate analysis, participants who reported familiarity had a slightly higher score in the barriers domain than those who did not (2.21 vs. 2.13, p=.013). Since higher scores represent fewer perceived barriers, the familiar group reported somewhat a lower burden of barriers. However, this domain was not retained in the final domain-level logistic regression model after the other questionnaire domains were considered. Thus, perceived barriers differed between the two groups in the unadjusted comparison but did not show an independent association with self-reported familiarity in the multivariable analysis. This doesn't mean that barriers are not important but rather reflects their role as modifying factors between knowledge and actions, not determinants of knowledge itself. This is consistent and aligns with The Health Belief Model (25). These findings suggest that perceived barriers and self-reported familiarity are related at the bivariate level, although barriers did not show an independent domain-level association after the other questionnaire dimensions were considered. Their role may therefore be more complex than a direct relationship with familiarity and warrants further investigation. Therefore, working to overcome these obstacles, such as providing financial support, establishing mobile eye clinics, or implementing programs to bridge the trust gap between parents and healthcare providers, is important, but it is insufficient on its own if it is not accompanied by programs to increase parents' knowledge and awareness. The findings therefore suggest that both knowledge-related gaps and perceived access barriers warrant further attention, but their effects on actual care-seeking behavior cannot be determined from the present survey. Future studies should examine how these factors interact prospectively when parents make decisions about seeking pediatric eye care.

### Who responded and what that means for generalizability

The characteristics of the study sample should be considered when interpreting the findings. Most participants were women, which likely reflects both a greater willingness to take part in health-related surveys and the prominent role mothers often play in matters related to children's health in Jordan. The sample was also relatively well educated, with more than 73% of participants reporting a diploma or higher qualification. In addition, most respondents lived in urban areas, and a substantial proportion reported that at least one of their children had previously been diagnosed with an eye disorder.

Recruitment through social media introduces an additional possibility of selection bias. Parents who regularly use digital platforms, are more comfortable completing online questionnaires, or have a particular interest in children's health may have been more likely to participate. Parents with limited internet access, lower digital engagement, or less interest in health-related topics may consequently be underrepresented. Although participants were recruited from all 12 governorates, broad geographic coverage does not make the sample nationally representative because participation was voluntary and was not based on probability sampling.

Taken together, these features suggest that the sample may represent a somewhat more health-aware and socially advantaged group than the general population. As a result, the level of parental familiarity observed in this study may be somewhat higher than what might be expected in the wider Jordanian community. This makes the gaps identified in knowledge and treatment-related understanding even more important, since they were observed in a group that might reasonably be expected to have better access to information and care than many others.

### Limitations

Several limitations should be considered when interpreting the findings of this study. First, the cross-sectional design does not allow conclusions to be drawn about causality or the direction of the relationships observed between familiarity and the other study variables.

Second, the study relied on self-reported responses, which introduces the possibility of social desirability bias. Some participants may have provided answers they believed were more appropriate or acceptable rather than responses that fully reflected their actual knowledge or beliefs. Using a single binary question to measure self-reported familiarity may not accurately represent a parent's actual knowledge of pediatric eye disorders. Participants could have overestimated their familiarity because of confidence or social desirability. Others may have underestimated it despite answering knowledge items correctly. There is also conceptual overlap between the self-reported familiarity outcome and the domains assessing knowledge of pediatric eye diseases and treatment-related understanding. Consequently, associations between these domains and familiarity should not be interpreted as evidence that they predict an independent behavioral or objective knowledge outcome. Rather, they indicate correspondence between participants' perceived familiarity and their responses to related questionnaire content. Although the adjusted analyses used education, medical background, previous diagnosis of an eye disorder in a child, income, sex, and residence, other factors influencing perceived familiarity, such as confidence or prior exposure to eye-health information, were not directly measured. Future studies could combine self-reported familiarity with a validated knowledge test, clinical scenarios, or an independently defined knowledge threshold.

The use of voluntary social-media recruitment may also have introduced selection bias. Parents who were more digitally engaged, better educated, more interested in health issues, or who had previous experience with childhood eye problems may have been more likely to participate. Women and urban and highly educated parents were consequently overrepresented in the sample. Although participants came from all 12 governorates, the sampling method was not probability-based. Therefore, the sample should not be considered nationally representative. It is also possible that the proportion reporting familiarity was higher than would be observed in the wider population of Jordanian parents.

The questionnaire did not record the exact caregiving relationship of respondents who reported having no children under 18 years of age. It is therefore not possible to determine whether these participants were grandparents, legal guardians, or other caregivers. Future studies should record the caregiver's relationship to the child more explicitly.

The three-point Likert scale is another limitation to consider. This format was retained after pilot testing because participants found it easier to understand and complete, but it offers less variation than a five- or seven-point scale. As a result, small differences between participants may have been less clearly captured. The domain scores should therefore be interpreted as reflecting broad patterns of agreement, neutrality, and disagreement rather than finely graded differences in opinion. The wording and scoring direction of Domain 5 also require some caution. Because lower scores represented greater perceived barriers, the direction of the scale may be less intuitive than in the other domains and could complicate interpretation if not clearly explained.

Finally, backward elimination was used at the item level as an exploratory screening method to reduce the original set of questionnaire items to a smaller group of candidate variables. Stepwise procedures are known to be sensitive to the particular sample being analyzed and may produce overly optimistic estimates. For this reason, the selected items should be interpreted as exploratory findings rather than as a definitive set of predictors. Although the other items were subsequently examined in an adjusted forced-entry model, both stages were conducted using the same sample. The stability and predictive performance of these item-level findings should therefore be assessed in an independent dataset.

## Conclusion

This study characterized several dimensions of pediatric eye-health awareness among participating parents in Jordan and identified factors associated with their self-reported familiarity with common pediatric eye disorders. Parents generally expressed positive attitudes toward professional eye care, while gaps remained in knowledge of specific diseases and treatment-related issues. Knowledge of pediatric eye diseases and understanding of related treatments were among the factors most consistently associated with self-reported familiarity, and several specific knowledge and treatment items remained associated after adjustment for participant characteristics. Although participants reporting familiarity had higher scores across all five domains in the bivariate comparisons, only knowledge of pediatric eye diseases and treatment-related understanding remained independently associated with familiarity in the domain-level regression.

These associations should not be taken to mean that familiarity or knowledge directly leads parents to seek care, accept treatment, follow medical advice, or obtain earlier assessment for their children. These behaviors were not measured in this cross-sectional survey. Instead, the findings point to specific areas of parental knowledge and perception that deserve further attention in future educational work and in prospective studies that directly examine care-seeking behavior.

The item-level results also identify several topics that could be emphasized in future parental education. These include the age-sensitive nature of amblyopia, its association with strabismus, and common misconceptions about prescribed eyeglasses. Such findings may help guide the content of future educational programs, but whether these programs improve objective knowledge or influence healthcare behavior will need to be tested directly.

The study also identified perceived financial, logistical, and trust-related barriers to pediatric eye care. Financial concerns were particularly prominent and included the costs of clinic visits, eyeglasses, and surgical treatment. These findings may be useful when considering future service-planning or educational strategies, including ways to improve access to reliable eye-health information and reduce practical barriers to care. However, this study did not evaluate the effectiveness or cost-effectiveness of any specific educational, screening, or healthcare intervention.

Overall, the findings provide a clearer picture of the areas in which parental knowledge, perceptions, and perceived barriers may be most relevant to pediatric eye health in Jordan. They may help guide future research and the development of educational or service-based initiatives. However, before making specific national policy recommendations, population-based studies and prospective evaluations of proposed interventions are still needed.

## Data Availability

The datasets presented in this article are not readily available because data summary and analysis outcomes are available by request to the corresponding author. Requests to access the datasets should be directed to Asma Musleh, asma.musleh@bau.edu.jo.

## References

[1] Loh L Prem-Senthil M Constable PA. A systematic review of the impact of childhood vision impairment on reading and literacy in education. J Optom. (2024) 17(2):100495. 10.1016/j.optom.2023.100495 37918059 PMC10641537

[2] World Health Organization. World Report on Vision. Geneva: World Health Organization (2019).

[3] International Agency for the Prevention of Blindness. Child eye health (2024).

[4] Pascolini D Mariotti SP. Global estimates of visual impairment: 2010. Br J Ophthalmol. (2012) 96(5):614–8. 10.1136/bjophthalmol-2011-300539 22133988

[5] Hashemi H Fotouhi A Yekta A Pakzad R Ostadimoghaddam H Khabazkhoob M. Global and regional estimates of prevalence of refractive errors: systematic review and meta-analysis. J Curr Ophthalmol. (2018) 30(1):3–22. 10.1016/j.joco.2017.08.009 29564404 PMC5859285

[6] Vision Center. Statistics on pediatric eye health (2025).

[7] Cotter SA Edwards AR Wallace DK Beck RW Arnold RW Astle WF. Treatment of anisometropic amblyopia in children with refractive correction. Ophthalmology. (2006) 113(6):895–903. 10.1016/j.ophtha.2006.01.068 16751032 PMC1790727

[8] Rajavi Z Khorrame Z Ashrafi S. The effects of refractive status on the outcomes of strabismus surgery in patients with esotropia. BMC Ophthalmol. (2024) 24:271. 10.1186/s12886-024-03531-5 38918731 PMC11197173

[9] Sherief ST Tesfaye S Eshetu Z Ali A Dimaras H. Exploring the knowledge, attitudes, and practice towards child eye health: a qualitative analysis of parent experience focus groups. PLoS One. (2023) 18(11):e0293595. 10.1371/journal.pone.0293595 37922264 PMC10624311

[10] Morrison AK Glick A Yin HS. Health literacy: implications for child health. Pediatr Rev. (2019) 40(6):263–77. 10.1542/pir.2018-0027 31152099

[11] Masarwa D Niazov Y Ben Natan M Mostovoy D. The role of parental health beliefs in seeking an eye examination for their child. BMC Ophthalmol. (2023) 23:269. 10.1186/s12886-023-02994-2 37312052 PMC10262523

[12] Almogbel AH Al Shanbari N Alibrahim IS Alsaadi SS Algarni HS Alshanbari AS. Parents’ awareness and attitude toward pediatrics eye diseases in makkah, Saudi Arabia: a cross-sectional study. Cureus. (2023) 15(5):e38366. 10.7759/cureus.38366 37265878 PMC10230266

[13] Surrati AM Almuwarraee SM Mohammad RA Almatrafi SA Murshid SA Khayat LI. Parents’ awareness and perception of children’s eye diseases in Madinah, Saudi Arabia: a cross-sectional study. Cureus. (2022) 14(2):e22604. 10.7759/cureus.22604 35371836 PMC8958142

[14] Alghamdi MA Almustanyir A Alshuimi AA Alrasheed SH Alabdulkader B Alanazi M. Prioritizing pediatric eye care in Saudi Arabia: a national Delphi consensus study. Healthcare. (2025) 13(19):2467. 10.3390/healthcare13192467 41095553 PMC12523716

[15] Haddad MF Bakkar MM Abdo N. Public awareness of common eye diseases in Jordan. BMC Ophthalmol. (2017) 17:177. 10.1186/s12886-017-0575-3 28969614 PMC5625650

[16] Ababneh LT Khriesat W Dalu SA Hanania RJ Ababneh BF Bany Amer NA. Knowledge of and attitude to eye disorders among pediatricians in north Jordan. Ann Med Surg. (2021) 67:102430. 10.1016/j.amsu.2021.102430 PMC8209081 34168867

[17] Setia MS. Methodology series module 3: cross-sectional studies. Indian J Dermatol. (2016) 61(3):261–4. 10.4103/0019-5154.182410 27293245 PMC4885177

[18] Eysenbach G. Improving the quality of web surveys: the checklist for reporting results of internet E-surveys (CHERRIES). J Med Internet Res. (2004) 6(3):e34. 10.2196/jmir.6.3.e34 15471760 PMC1550605

[19] Faul F Erdfelder E Lang AG Buchner A. G*power 3: a flexible statistical power analysis program for the social, behavioral, and biomedical sciences. Behav Res Methods. (2007) 39(2):175–91. 10.3758/BF03193146 17695343

[20] Alanazi SRG Alanazi HWH Alanazi WG Alanazi NSQ Alenezi DOB Al-Sweilem M. Parents’ knowledge and attitudes toward pediatric ophthalmic disorders in Saudi Arabia: a cross-sectional study. Pediatr Rep. (2024) 16(4):902–20. 10.3390/pediatric16040077 39449404 PMC11503256

[21] Alsaqr AM. Eye care in young children: a parents’ perspective of access and barriers. J Ophthalmic Vis Res. (2023) 18(2):192–201. 10.18502/jovr.v18i2.13186 37181614 PMC10172806

[22] Podsakoff PM MacKenzie SB Lee JY Podsakoff NP. Common method biases in behavioral research: a critical review of the literature and recommended remedies. J Appl Psychol. (2003) 88(5):879–903. 10.1037/0021-9010.88.5.879 14516251

[23] Field A. Discovering Statistics Using IBM SPSS Statistics. 5th ed. London: SAGE Publications (2018).

[24] Al Musalami SN Al Qasim RJ Alshuhayb BS Al-Somali AI. Insights into parental perspectives: children’s eye care in Saudi Arabia. Heliyon. (2025) 11(1):e41179. 10.1016/j.heliyon.2024.e41179 39811275 PMC11730857

[25] Rosenstock IM. Historical origins of the health belief model. Health Educ Monogr. (1974) 2(4):328–35. 10.1177/109019817400200403 299611

