# Supplementary material for: Factors associated with parents' self-reported familiarity with pediatric eye disorders in Jordan: a quantitative analysis of survey data from Jordanian parents
Source: Front Med (Lausanne). 2026 Sep 11;13:1935571. doi: 10.3389/fmed.2026.1935571 (PMC13612285; doi:10.3389/fmed.2026.1935571)
Supplement: Supplementary file 1 [file Datasheet1.docx]

0. **Demographics:**
1. Gender: (Male, Female)
2. Age:
3. Marital Status: (Married, Divorced, Widow)
4. Province: (Amman, Irbid, Zarqa, Ajlun, Jerash, Karak, Ma’an, Madaba, Mafraq, Aqaba)
5. Area of residence: (Rural, Urban)
6. Educational Level: (No education, Elementary School, Middle school, High School, Diploma, Bachelor, Master, PhD)
7. Employment Status: (Student, Medical field worker, Government employee, Private employee, Business owner, Retired, Unemployed)
8. Monthly Income: (<500 JD, 500-1000JD, 1000-2000JD, >2000JD)
9. No. of Children (under 18 years old) (1-2, 3-4, 5-6, >6, No children)
10. Has any of your children been diagnosed with eye disorder? Yes	No
1. **Domain 0: Source of Knowledge About Pediatric Eye Disorders:**

1. Are you familiar with common pediatric eye disorders:

(1= Yes, 2 = No)

2. **Domain 1: Parents' knowledge of pediatric eye disease:**

Rate your agreement with the following statements: *(1 = Disagree, 2=Neutral, 3 = Agree)*

1. Amblyopia (lazy eye) which is reduced vision in the eye due to abnormal development of vision can affect children up to 7-8 years old:
2. A child could have a refractive error (nearsightedness, long-sightedness and astigmatism) without complaining of blurry vision
3. Delay in treatment of refractive errors could lead to amblyopia:
4. Strabismus (eye misalignment) in children could lead to amblyopia:
5. It is *ab*normal to see strabismus in a child up to 1 year old:
6. Eye surgeries, when recommended, are necessary for children’s vision health.
3. **Domain 2: Recognition of Eye Disease Symptoms and Causes**

Rate your agreement with the following statements: *(1 = Disagree, 2=Neutral, 3 = Agree)*

1. Children who struggle to copy from the school board or perform poorly in school should have an eye examination.
2. Headaches in children can result from eye problems.
3. Frequent rubbing of the eye indicates an eye problem:
4. Poor focus in a child could be due to an eye problem.
5. Frequent blinking, light sensitivity and squeezing of the eyes in a child are indications of having an eye exam:
6. Eye pain, watering and discharge are not always caused by an eye infection.
7. Difficulty identifying colors in a child could indicate an eye disorder.
8. Tilting the head frequently could indicate an eye condition.
9. Avoiding close objects or toys might indicate an eye problem in a child.
4. **Domain 3: Treatment and Management of Eye Disorders**

Rate your agreement with the following statements: *(1 = Disagree, 2=Neutral, 3 = Agree)*

1. Wearing prescribed glasses helps children develop normal vision:
2. Wearing glasses all the time does not make children dependent on them and does not worsen their vision.
3. Amblyopia can be treated depending on the child's age.
4. Amblyopia can be treated by surgeries and/or glasses and/or eye drops with covering the good eye
5. It is not true to ignore lacrimal obstruction until school age.
6. Treatment of strabismus includes glasses and surgery:
7. Correcting strabismus surgically does not eliminate the need to wear glasses
8. Using eye drops without consulting a doctor is risky for children.
9. Strabismus in children is not treated for cosmetic reasons only:
10. Eye disorders like cataract and glaucoma could affect old people and children:
5. **Domian 4: Parental Attitudes Towards Eye Health and Care**

Rate your agreement with the following statements: *(1 = Disagree, 2=Neutral, 3 = Agree)*

1. All children should have a routine eye exam even if they show no signs of vision problems:
2. The first routine eye exam of the child with no vision complaints should be done at the age of 3-5 years:
3. School vision screening is not sufficient to detect all vision problems in children:
4. A child with a family history of pediatric eye disorders should have an eye examination with an ophthalmologist even if they don’t complain about their vision:
5. Regular eye exams every 6-12 months are necessary for the children’s eye health:
6. Parents should follow the eye doctor’s recommended treatment or prescribed glasses even if they believe their child doesn’t need them:
7. If the child’s teacher or pediatrician recommends an eye examination, parents should follow their recommendation
8. Previously prescribed eye drops to one child in a family shouldn’t be used on another child in the same family without consulting the eye doctor:
9. Using electronic devices and sitting in front of TV screens for a long time increases the possibility of the child suffering from myopia, dryness and eye strain
6. **Domain 5: Barriers to Eye Care**

*Note on scoring and presentation:*
The questionnaire was originally administered to participants in Arabic. In the original Arabic questionnaire, the Domain 5 items were worded so that agreement indicated a greater perceived barrier to pediatric eye care; a direct English translation would retain this direction. For the analyses reported in the present study, all eight Domain 5 items were reverse-scored so that higher scores represented fewer perceived barriers and lower scores represented greater perceived barriers. On the three-point response scale, responses were recoded as 1 → 3, 2 → 2, and 3 → 1. Accordingly, the English wording of the Domain 5 items is expressed in the corresponding reverse direction, in parallel with the reverse scoring described above, so that the wording and numerical coding remain consistent. This transformation changed only the direction in which the responses were scored and presented and did not alter the substantive information provided by any participant.

Rate your agreement with the following statements: *(1 = Disagree, 2=Neutral, 3 = Agree)*

1. Financial constraints, such as doctor’s fees and the cost of glasses or surgery, do not prevent me from getting eye care for my child.
2. The distance to the nearest eye doctor or lack of transportation does not make it difficult to access eye care for my child.
3. The need for several doctor visits and longer waiting times in the clinic does not affect my decision and commitment to do an eye test for my child
4. Difficulties in eye testing, like using eye drops or using eye clamps to open eyelids, may not affect my decisions regarding my child's eye health
5. Concerns about the cosmetic appearance of wearing glasses or my child being bullied by his peers do not discourage me from seeking care for my child.
6. Concerns about the outcomes of eye surgery or the complications of general anesthesia may not affect my agreement to any surgical intervention recommended by doctor.
7. Recommendations and advice from family and friends can not affect my decisions regarding my child’s eye health and care.
8. Lack of trust in health care providers and the level of services does not influence my decisions regarding my children's eye health.
